# Derivatization Ion Chromatography for the Determination of Monoethanolamine in Presence of Hydrazine in PHWR Steam-Water Circuits

**DOI:** 10.1155/2011/813061

**Published:** 2011-07-12

**Authors:** Ayushi D., Arijit Sengupta, Sangita D. Kumar, A. G. Kumbhar, G. Venkateswaran

**Affiliations:** ^1^Analytical Chemistry Division, Bhabha Atomic Research Centre, Mumbai 400085, India; ^2^Radiochemistry Division, Bhabha Atomic Research Centre, Mumbai 400085, India; ^3^Water and Steam Chemistry Division, Bhabha Atomic Research Centre, Mumbai 400085, India

## Abstract

A simple, rapid and accurate method for the determination of monoethanolamine (MEA) in PHWR steam-water circuits has been developed. MEA is added in the feed water to provide protection against corrosion while hydrazine is added to scavenge dissolved oxygen. The quantitative determination of MEA in presence of hydrazine was accomplished using derivatization ion chromatography with conductometric detection in nonsuppressed mode. A Metrosep cation 1-2 analytical column and a Metrosep cartridge were used for cation separation. A mixture of 4 mM tartaric acid, 20% acetone and 0.05 mM HNO_3_ was used as eluent. Acetone in the mobile phase leads to the formation of different derivatives with MEA and hydrazine. The interferences due Na^+^ and NH_4_^+^ were eliminated by adopting a simple pretreatment procedure employing OnGuard-H cartridge. The limit of detection limit of MEA was 0.1 **μ**g mL^−1^ and the relative standard deviation was 2% for the overall method. The recovery of MEA added was in the range 95%–102%. The method was applied to the determination of MEA in steam generator water samples.

## 1. Introduction

In Indian pressurized heavy water reactors (IPHWRs), the nuclear heat is transported by the heavy water (D_2_O) coolant in the primary heat transport system to the secondary light water (H_2_O) for steam production. In the steam-water circuit, two-phase erosion corrosion is a serious operational issue. To provide protection against the corrosion, volatile amines are added in the feed water of steam generators (SG) to raise the pH of the water. These amines volatilize along with the steam and are partly carried away to the turbine and condenser part, thereby providing protection against corrosion to the entire steam-water circuit. Along with amine, hydrazine is injected to scavenge dissolved oxygen, and thereby produce reducing conditions. The treatment is called as an all volatile treatment (AVT), and the amines are used as AVT reagents [1–3]. Ammonia (earlier used) was replaced by cyclohexylamine and morpholine as AVT reagents because of their less volatility [3, 4]. This change allowed the pH of the steam condensate from the turbine cycle to be increased to a level that significantly reduced flow-assisted corrosion. MEA, because of its less volatility and higher base strength, has eventually replaced cyclohexylamine and morpholine [5–7]. 

Quantification of MEA is an essential step in determining its appropriate amount to use for maximal protection. The accurate and reliable determination of MEA in the presence of hydrazine is a challenging analytical problem because of their similar chemical properties. The spectrophotometric method developed in our laboratory earlier could not be applied for the determination of MEA because of severe interference from hydrazine [4]. There are a few reports on the determination of MEA in environmental, oil and gas industry water samples [8–14]. However, there is not a single report in the literature on the determination of MEA in the presence of hydrazine in the steam generator water. The Metrohm application note C107 for the determination of MEA and hydrazine is not applicable for steam-generator water which contains several cationic impurities along with these two additives [11].

Ion chromatography (IC) finds wide applications in power plant industry for the determination of trace concentrations of ionic impurities in feed and process streams [15–18]. IC is an excellent technique for the separation of anions and cations of closely related structures. The aim of the present study was to use this technique for developing a specific, interference-free, and sensitive analytical method for the detection of MEA in PHWR steam-water circuits. The method is based on a chemical conversion of the two compounds—MEA and hydrazine by reaction with acetone which is contained in the eluent. A one-step sample pretreatment procedure was adopted to eliminate the interference of alkali metal ions. Using this procedure, it was possible to directly analyze the MEA by ion chromatography in non-suppressed mode using a mixture of tartaric acid, acetone and HNO_3_ acid as eluent and conductometric method of detection. The method was applied to several steam generator water samples.

## 2. Experimental

### 2.1. Instrumentation

Ion chromatography was performed with Metrohm (Herisau, Switzerland) instrumentation consisting of a 709 IC Pump, 733 IC Separation Center and a 732 IC conductivity detector. Cation separation was carried out in nonsuppressor mode on a Metrosep cation 1-2 analytical column (125 × 4 mm) connected in series with a Metrosep cartridge. The Metrosep cartridge is used as a precolumn to protect the analytical column.

### 2.2. Reagents

Reagents used were of analytical grade and obtained from Sarabhai M. Chemicals, Baroda, India. Deionised water (18 MΩ) obtained from a Barnstead water purification system (Barnstead, Boston, Mass, USA) was employed throughout. OnGuard-H cartridge was obtained from Dionex (Sunnyvale, Calif, USA). Stock standard solutions (1 g L^−1^) were prepared for MEA and hydrazine. The working standard mixtures for calibration were prepared weekly from the stock standard.

### 2.3. Ion Chromatographic Conditions

Different eluents were tested for the separation of MEA and hydrazine because both coeluted from the analytical column (Table 1). In each case, three different compositions (0.5, 1, 4 mM) and three different flow rates (0.8, 1.0, 1.2 mL min^−1^) were employed. Dipicolinic acid (pyridine-2,6 dicarboxylic acid) was added to remove the interference of transition metal by chelation in the sample solutions. The studies showed that it was possible to separate the two coeluting peaks with a mixture of 4 mM tartaric acid, 20% acetone and 0.05 mM HNO_3_. The injection volume was 20 *μ*L. An eluent flow rate of 0.8 mL min^−1^ was applied throughout. The acetone and HNO_3_ acid eluent were degassed for 5–10 minutes to remove air bubbles.

### 2.4. Sample Pretreatment

Steam generator water sample was passed through OnGuard-H cartridge to remove interfering cations (alkali and transition metal ions). The recommended guidelines for the use of OnGuard cartridges supplied by Dionex Corp. (Sunnyvale, Calif, USA) were followed. OnGuard-H cartridge was washed with 10 mL deionised water. About 5 mL of the sample solution was loaded on the cartridge and allowed to flow at the rate of 2 mL min^−1^. The first 3 mL of elute was rejected and the next 2 mL was collected. Before injection into the ion chromatograph, sample solutions were diluted (1 : 1) with mobile phase in order to maintain the same pH. This step also ensures that amines are in the protonated form. 

## 3. Results and Discussion

### 3.1. Separation of MEA and Hydrazine Using Derivatization Ion Chromatography

**Figure 1:**
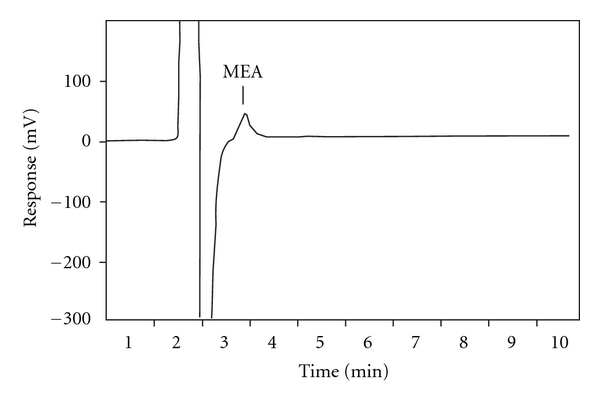
Chromatogram of a synthetic mixture of MEA (2 *μ*g mL^−1^) and hydrazine (20 *μ*g mL^−1^) showing only the MEA peak resolved from the large injection peak.

**Scheme 1:**
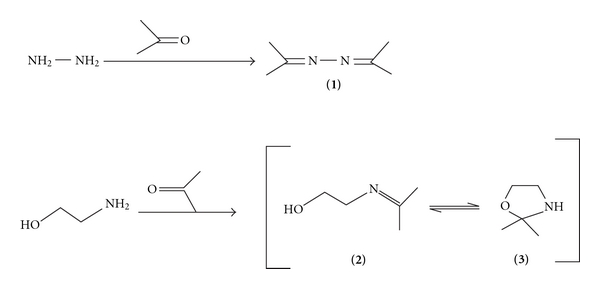


Due to their similar chemical properties, both MEA and hydrazine coeluted from the analytical column (Table 1). Hence, acetone was added to the eluent in order to derivatize the two components viz., MEA and hydrazine as shown in Scheme 1. Under these conditions of elution, no peak was obtained for hydrazine (20 *μ*g mL^−1^), and the peak due to MEA (2 *μ*g mL^−1^) was obtained at a retention time of 2.81 min (Figure 1). Thus, using this mobile phase, determination of MEA was possible without any interference from hydrazine. 

The different selectivities of MEA and hydrazine for the stationary phase and hence their retention times on the column using this mobile phase could be explained by the rapid reactions of hydrazine and MEA with acetone. It is well known that simple mixing of amine and carbonyl compound at room temperature produces an imine, and in fact, the reaction is so vigorous in the case of hydrazine that not only the reaction mixture needs to be cooled in an ice bath, but also the rate of addition of hydrazine is controlled [19]. Hence, under the elution conditions (i.e., very high concentration of acetone 20%), it is expected that the reaction of these amines goes to completion giving rise to acetone azine (**1**) [20] and imine (**2**) [19], respectively. 

Thus, in the case of hydrazine, the acetone azine (**1**) formed eluted in the injection peak while in the case of MEA, imine (**2**), and its ring tautomer (**3**) were formed [19] and it eluted at a retention time of 2.81 min.

The aqueous elution media optimized in our study contained 100 fold dilute inorganic acid (0.05 mM HNO_3_) as compared to that used in the Metrohm application note C107 [11]. This is of great advantage in terms of enhanced sensitivity of the conductivity detector and milder conditions of operation for the instrumentation as well as the analyst. 

### 3.2. Analytical Method for Determination of MEA

A linear calibration plot with a regression coefficient (*r* ^2^) of 0.9998 was obtained for standard MEA solution in the range of 0.3–50 *μ*g mL^−1^. The relative standard deviation evaluated by replicate analysis (*n* = 6) was 2%. The limit of detection was 0.1 *μ*g mL^−1^ (*S*/*N* = 3) and limit of quantification was 0.3 *μ*g mL^−1^ (*S*/*N* = 10). 

### 3.3. Determination of MEA in Steam Generator Water Samples

Typical water chemistry specifications in the secondary system of PHWR are given in Table 2. Thus, along with MEA and hydrazine, Na^+^, Ca^+2^, Mg^+2^, and NH_4_^+^ are present as cationic impurities. The studies showed that Na^+^ and NH_4_^+^ ions in the sample matrix coelute with MEA peak. In order to overcome these difficulties, a simple and rapid pretreatment procedure was carried out using OnGuard-H cartridge. It contains styrene-based, strong acid resin in the H^+^ form. The cation exchange capacity is 2–2.5 meq/cartridge. It was used for the removal of alkali, alkaline earths, transition metals, and NH_4_^+^ from the sample matrix. The selective removal of the interfering cations leaving the MEA in solution was tested by the recovery experiments carried out at different concentration levels of MEA. The MEA solutions were spiked with different cationic impurities (10 *μ*g mL^−1^) before pretreatment. The studies showed that no peak could be observed for the different cations added. Further, the recoveries of MEA were in the range of 95%–102%. Thus, the pretreatment procedure selectively removed the interfering cations (Table 3).

**Figure 2:**
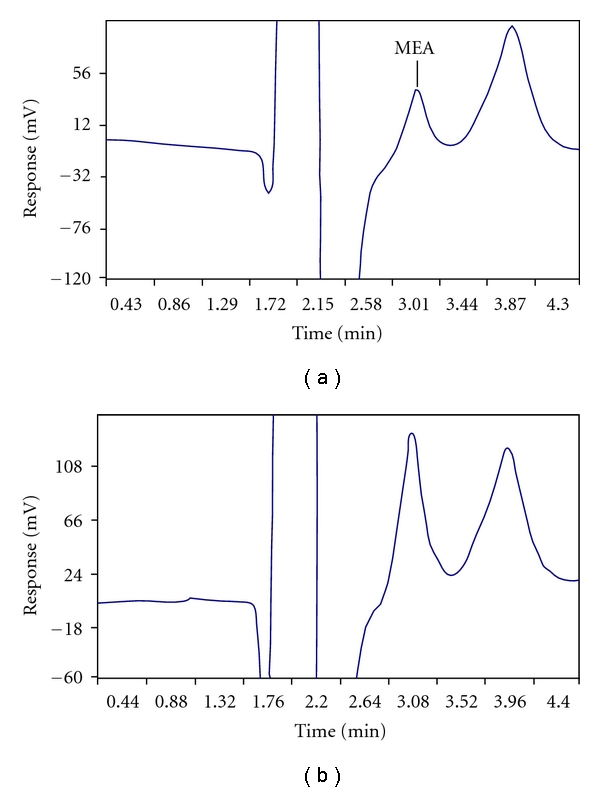
Chromatogram of steam generator water samples showing (a) MEA peak and an impurity peak (b) enhancement of the MEA peak on spiking the sample solution with MEA (5 *μ*g mL^−1^).

As samples were diluted 1 : 1 with mobile phase, a chromatogram of this blank solution was recorded in order to find out the contribution from impurities, if any, present in mobile phase. There was no peak observed for MEA or other cations in the blank solution. A typical chromatogram of steam generator (SG) water sample is shown in Figure 2(a). It is seen that the MEA peak (*R*_*t*_ = 2.8 min) is well separated from the injection peak and another peak at retention time *R*_*t*_ = 3.76 min, which was due to some impurity in SG water. MEA peak in the sample was also confirmed by the enhancement of the peak on spiking with the MEA standard solution (5 *μ*g mL^−1^) (Figure 2(b)). Ion chromatographic analysis of MEA in different SG water samples received from power plant was carried out. The MEA content was found to be in the concentration range of 4–8 *μ*g mL^−1^.

## 4. Conclusion

The derivatization ion chromatographic method developed for MEA determination in the presence of hydrazine has been shown to be sensitive and specific. The required sensitivity and precision were achieved by the use of acetone in the mobile phase, a single-step pretreatment, low conductivity, and stable baseline. The technique was applied to the determination of MEA in steam generator water from the power plant. For solutions having high salt concentration, a single OnGuard-H may not be enough, and also the recovery of MEA needs to be carried out before the application of this method.

## Figures and Tables

**Table 1:** Retention time (*R*_*t*_) studies of MEA and hydrazine in different mobile phase.

Composition of mobile phase	MEA *R*_*t*_ min	Hydrazine *R*_*t*_ min
4 mM tartaric acid + 1 mM dipicolinic acid	2.37	2.38
1 mM oxalic acid	4.64	4.66
4 mM tartaric acid + 20% acetone + 0.05 mM HNO_3_	2.81	No peak

**Table 2:** Typical water chemistry specifications in the secondary cycle of steam-generating systems: MEA, hydrazine and ionic impurities.

Parameter	Permissible range
MEA, *μ*g mL^−1^	3–7
Hydrazine, *μ*g L^−1^	50–200
pH	9-10
NH_4_^+^, *μ*g mL^−1^	<1
Na^+^, *μ*g L^−1^	<5
*SO*_4_^−2^, *μ*g mL^−1^	<1
Total Hardness, *μ*g mL^−1^	<2
Silica, *μ*g L^−1^	<20

**Table 3:** Recovery of MEA in sample.

Added (*μ*g mL^−1^)	Found (*μ*g mL^−1^)	% Recovery
1	0.95	95
5	5.1	102
10	9.8	98
